# Retroperitoneal Ganglioneuroma Encasing the Celiac and Superior Mesenteric Arteries

**DOI:** 10.1100/tsw.2004.198

**Published:** 2004-11-18

**Authors:** Justin K. Nelms, Eric K. Diner, Ernest E. Lack, Sunil V. Patel, Seyed R. Ghasemian, Mohan Verghese

**Affiliations:** Department of Urology, Washington Hospital Center, Washington, DC, USA

**Keywords:** ganglioneuroma, adrenal glands, neoplasm, laparoscopy

## Abstract

Ganglioneuroma is a rare neoplasm arising from the sympathoadrenal neuroendocrine system and has anatomic distribution paralleling the sympathetic chain ganglia and the adrenal medulla. In some cases, ganglioneuroma is the end stage maturation of less-differentiated neoplasms such as neuroblastoma or ganglioneuroblastoma, but based on age at diagnosis (over 10 years of age) and anatomic location, many of these tumors appear to arise *de novo*. It must be included in the differential diagnosis of posterior mediastinal and retroperitoneal mass. We report a case of retroperitoneal ganglioneuroma involving the celiac axis and superior mesenteric arteries in a 40-year-old female.

## INTRODUCTION

Ganglioneuroma is a rare neoplasm arising from the sympathoadrenal neuroendocrine system and has anatomic distribution paralleling the sympathetic chain ganglia and the adrenal medulla. In some cases, ganglioneuroma is the final stage of maturation of less-differentiated neoplasms such as neuroblastoma or ganglioneuroblastoma, but based on age at diagnosis (over 10 years of age) and anatomic location, many of these tumors appear to arise *de novo*. It must be included in the differential diagnosis of posterior mediastinal and retroperitoneal mass. We report a case of retroperitoneal ganglioneuroma involving the celiac axis and superior mesenteric arteries in a 40-year-old female.

## CASE REPORT

A 40-year-old female presented to her primary physician with intermittent abdominal pain of 1-year duration. The patient denied any weight change, hypertension, or genitourinary symptoms. She had no family history of cancer or other tumors. She denied tobacco use and her surgical history was significant only for cholecystectomy. Her review of systems and physical exam were normal, without hypertension or a palpable mass. An ultrasound revealed a left suprarenal mass, which was confirmed on MRI to be a left adrenal mass measuring 5.0 × 2.8 × 4.5 cm. Laboratory workup included normal blood count, serum electrolytes, cortisol, and aldosterone. The 24-h urinary levels of VMA and metanephrines were in the normal range. Two separate CT-guided, fine-needle aspirates of the mass revealed fibrous and mixed neural tissue of benign histology. She chose to observe the mass.

Five months later, an MRI revealed minimal size increase of the left suprarenal mass encasing the superior mesenteric artery (Fig. 1A) and she elected to have surgical resection. At laparoscopy, the adrenal gland was noted to be grossly normal and an adjacent mass was identified draping the aorta and encasing the celiac axis (Fig. 1B). The abdomen was then opened and the tumor (8.0 × 3.7 × 3.5 cm) was completely excised. Histopathological evaluation revealed a normal adrenal gland and a ganglioneuroma with multiple ganglion cells in a dense matrix of Schwann cells. There were no immature elements (Fig. 2). She recovered well postoperatively and will undergo annual MRI screening.

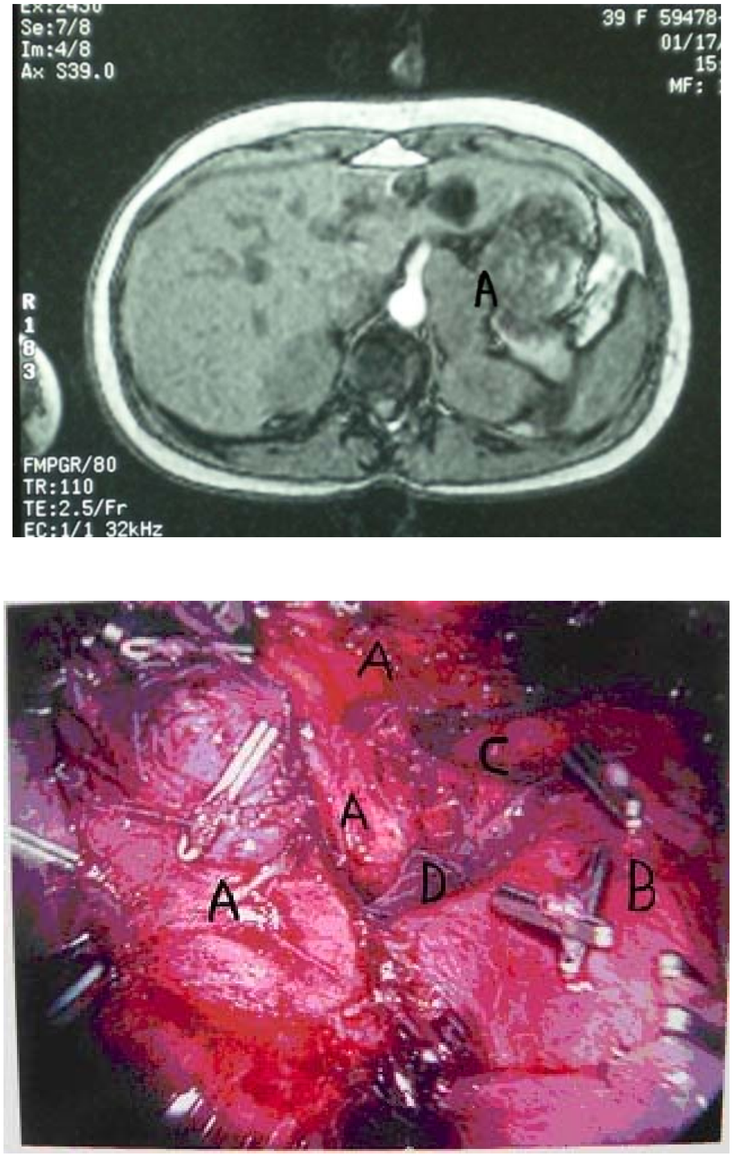

**FIGURE 1**. (**A**) MRI shows left suprarenal mass (A) encasing the superior mesenteric artery; (**B**), operative finding of left suprarenal mass (A) reflected off the aorta (B) , celiac axis (C) and aortic reflection (D).

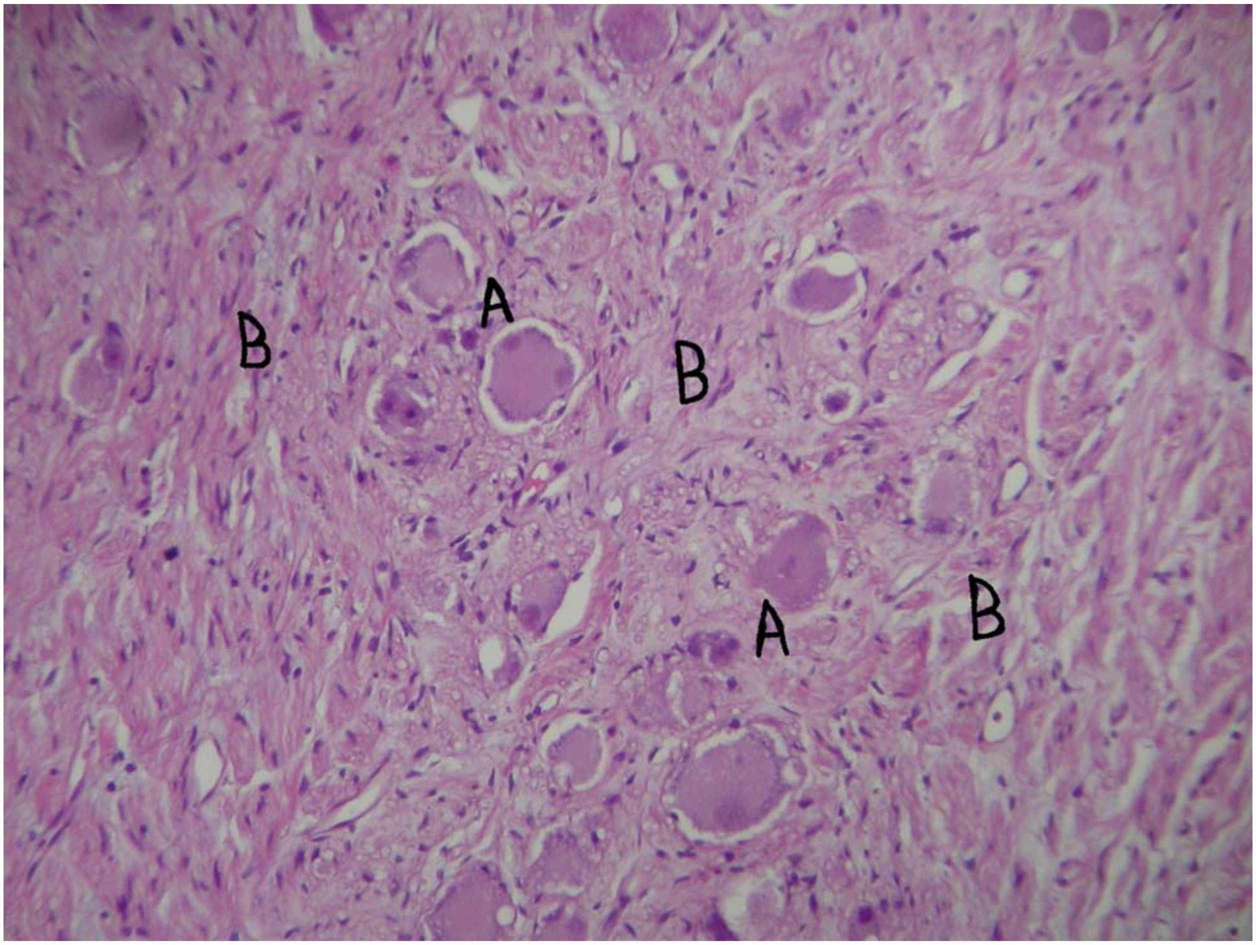

**FIGURE 2**. Irregular distribution of histologically mature ganglion cells (A) in a background of spindle cell matrix representing Schwann cells (B). H & E, reduced from ×200.

## DISCUSSION

Ganglioneuromas are rare, fully differentiated neoplasms, often making their diagnosis elusive. Most tumors are found incidentally, but patients may also present with abdominal pain or neurological symptoms due to compression of local structures. There have also been reports of ganglioneuromas with secretory activity such as increased catecholamine secretion or increased MIBG uptake, causing diarrhea, episodic hypertension, sweating, and flushing[1]. Diarrhea is associated with production of vasoactive intestinal polypeptide by ganglion cells. In younger patients, these factors can make clinical exclusion of neuroblastoma difficult, although it has been suggested that MR imaging can distinguish ganglioneuromas from neuroblastoma or pheochromocytoma[2]. There have also been rare reported cases of malignant degeneration of ganglioneuroma to neuroblastoma[3], making surgical excision and tissue investigation the management of choice.

Ganglioneuromas are usually found in the posterior mediastinum or retroperitoneum, particularly the presacral space, but may occur anywhere along the sympathetic chain. Retroperitoneal tumors are usually extra-adrenal. There is some debate as to whether ganglioneuromas arise *de novo* or if they represent an end product of maturing neuroblastoma and ganlioneuroblastomas. An interesting feature is the tendency to partially or completely surround blood vessels without compromising the lumen in most cases[3]. Current available data suggest an excellent prognosis and follow-up should include repeat imaging and monitoring for local recurrence or new symptomatology. The slow growing nature of these tumors also necessitates long-term follow-up.

## References

1. Geoerger, B., Hero, B., Harms, D., et al. (2001) Metabolic activity and clinical features of primary ganglioneuromas. *Cancer* **91(10),** 1905–1913.
2. Ichikawa, T., Koyama, A., Fujimoto, H., et al. (1993) Retroperitoneal ganglioneuroma extending across the midline: MR features. *Clin. Imaging* **17(1),** 19–21.
3. Randall, R., David, C., Goldfarb, H., et al. (1997) Adrenal and extra-adrenal retroperitoneal ganglioneuroma: imaging findings in 13 adults. *Radiology* **202(3),** 703–707.
